# The impact of prior knowledge on perceiving vocal elements in MIDI-converted music

**DOI:** 10.3389/fpsyg.2025.1565292

**Published:** 2025-09-30

**Authors:** Seth D. Metcalfe, Joseph A. Harris

**Affiliations:** Department of Psychology, Bradley University, Peoria, IL, United States

**Keywords:** perception, auditory, perceptual illusions, musical instrument digital interface, music

## Abstract

**Introduction:**

Illusions in which gaps in sensory evidence are filled in using prior knowledge represent a useful avenue for understanding the constructive nature of perception. The Musical Instrument Digital Interface (MIDI) vocals illusion, wherein listeners perceive the presence of vocal elements in a digitally converted audio format with none present, presents a novel opportunity to characterize the role of prior experience in auditory perceptual filling-in.

**Methods:**

In two experiments, participants reported the occurrence and duration of either imprecise or precise vocal elements in MIDI-converted audio. To isolate the effect of prior exposure on the emergence of the illusion in each experiment, the participants first listened to 12 MIDI-converted excerpts from a subset of six songs, with one half originally containing vocal elements and the others containing only instrumental tracks. Of the six songs, three were designated as “learned” and were presented in their original format during a subsequent learning block, and the remaining three were only presented in the MIDI format. This block sequence was repeated three times.

**Results:**

An imprecise perceptual illusion emerged regardless of prior exposure to original excerpts and distinguished between excerpts originally containing vocals and those containing only instrumental elements. A more precise illusory percept (words) emerged only for those MIDI stimuli corresponding to the original excerpts presented during the learning blocks.

**Discussion:**

These findings represent the first investigation of the MIDI vocals illusion and highlight distinct roles of bottom-up sensory features and top-down expectations based on experience in the perceptual filling-in of auditory information.

## 1 Introduction

### 1.1 Constructing perception

Perception is a constructive, computational process that fills explanatory gaps inherent to imperfect sensory signals through inferential processes (Helmholtz, 1866). The theoretical framework of predictive coding provides a useful set of testable principles that explain how the brain constructs subjective perceptual experience. Predictive coding posits that the brain maintains internal estimates of distal physical sources of sensory information and builds a useful percept by combining bottom-up sensory signals with top-down expectations about their nature (Gregory, 1980; Friston and Kiebel, 2009; Friston, 2012; Shipp, 2016). A comparison between bottom-up signals, driven by peripheral sensory information, and top-down signals yields a prediction error, which ascends through hierarchical cortical architecture to update the central representation via Bayesian inference, ultimately producing perception (Friston, 2010; Purves et al., 2001, 2011).

A key feature of a sensory/perceptual system that operates according to the principles of predictive coding is the influence of expectations on the interpretation of incomplete sensory information. One source of these expectations is the online learning of statistical regularities in the ongoing sensory stream. In the visual domain, this is well illustrated by the phenomenology of perceptual filling-in of blind spots. In the case of the retinal blind spot, an area of the retinal surface devoid of photoreceptors, the corresponding portion of the visual field is filled in with an estimate of the surrounding visual information, which can include static texture (Ramachandran and Gregory, 1991) and more complex features such as motion (Maus and Nijhawan, 2008). Similar surround-based perceptual filling-in is observed for purely perceptual blind spots, such as those associated with motion-induced blindness, wherein the awareness of a static parafoveal target superimposed on a globally moving array of distractors fluctuates (Bonneh et al., 2001; New and Scholl, 2008).

In addition to information gleaned from the statistical properties of ongoing sensory stimulation, top-down expectations are informed by long-term learning and prior experience. For example, pairs of visual objects are rated as more familiar if those objects had co-occurred during a prior passive viewing task (Fiser and Aslin, 2001). In the context of binocular rivalry, wherein incompatible simultaneous retinal inputs result in alternating perceptual dominance of those elements (Blake and Logothetis, 2002), prior exposure to one of the competing inputs results in it being more likely to initially dominate perception and maintain dominance for longer durations (Denison et al., 2011). This effect is also seen cross-modally, as a tone, previously associated with one of the two competing visual inputs, facilitates that input’s dominance when played during rivalry (Piazza et al., 2018).

### 1.2 Predictive processes and speech perception

In the auditory domain, predictive computational processes are essential, as many simultaneous streams must be parsed through auditory scene analysis of a single sensory input conflating multiple sources (Bregman, 1994). The extraction and reconstruction of speech content from degraded auditory input exemplify the auditory system’s use of bottom-up regularities in the sensory stimulus. For example, during the continuity illusion, wherein the auditory input interrupted by a brief gap of noise is perceived as continuous, fricative speech sounds tend to be perceived better than vowel sounds, and, more generally, the strength of this illusion depends on the similarity between the noise interruption and the original sound (Warren, 1970; Samuel, 1981; Riecke et al., 2008). Similarly, stimuli comprised of sinusoidal auditory signals following the formant center frequencies characteristic of human vocalizations, but without the harmonic spectra of human speech, are still perceived as words and sentences (Remez et al., 1981). In fact, spectrally degraded speech stimuli retaining only temporal cues characteristic of speech still evoke the accurate perception of words (Shannon et al., 1995). Noise-vocoded speech, used to simulate forms of sensorineural deafness treated through cochlear implants (Shannon, 1983; Zeng et al., 2008), consistently demonstrates the benefit of more elaborate bottom-up sensory information in the extraction of speech. The more complete these spectrally degraded signals are, the more intelligible the original speech signal becomes, with listeners showing better comprehension of noise-vocoded speech (Roberts et al., 2011; Souza and Rosen, 2009).

Top-down influences rooted in prior experience also serve the extraction of speech from degraded auditory stimuli. In the case of the continuity illusion, research utilizing word and pseudoword stimuli shows that the illusion is less likely to fail when noise bursts are embedded in words, suggesting that top-down template-based expectations support this repair process (Drożdżowicz, 2025; Shahin et al., 2009). In the case of noise-vocoded speech, cognitive factors, such as vocabulary, verbal learning, and recall abilities, predict higher intelligibility (Rosemann et al., 2017). In the short term, prior exposure and training with a set of noise-vocoded speech stimuli in one frequency region generalize to improved speech extraction from noise-vocoded speech with different frequency properties (Hervais-Adelman et al., 2011). In summary, the perception of speech relies on predictive coding mechanisms that support auditory scene analysis by (1) deriving predictions from ongoing sensory features and (2) leveraging expectations rooted in prior experience.

### 1.3 Expectations, prediction, and music perception

Music represents a distinct class of auditory stimulus, the perception of which is also determined by predictive processes incorporating bottom-up sensory information and top-down expectations (Narmour, 1991; Koelsch et al., 2019; Rohrmeier and Koelsch, 2012). Expectations and their violations are central to the multifaceted experience of music listening and manifest at multiple levels. For example, the mismatch negativity, a negative-polarity auditory-evoked potential whose amplitude scales with the dissimilarity of a current tone compared to the stream that preceded it, reflects a short-term auditory analysis process sensitive to violations of pitch expectations (Garrido et al., 2009). Expectations informed by online learning of statistical properties of music have implications for the perception of harmony, tone, key, and timbre, as well as qualities of rhythm (Pearce and Wiggins, 2006; Rohrmeier and Koelsch, 2012). Expectation violations also have implications for the aesthetic and emotional qualities of music and have been shown to affect physiological arousal and brain activity measures of reward processing (Steinbeis et al., 2006; Schultz et al., 1997). As with speech extraction, more long-term learning based on prior exposure provides expectations that inform the perception of music (Cheung et al., 2024). For example, while the detection of up-down pitch changes (contour coding) appears automatic, with expectation violations triggering the mismatch negativity in both naive and musically trained listeners, musically trained listeners show better detection of unexpected pitch distances (interval coding) accompanied by mismatch negativity (MMN) responses (Fujioka et al., 2004; Vuust et al., 2012).

### 1.4 The MIDI vocals illusion

The MIDI vocals illusion represents a novel and unique opportunity to further characterize the role of prior knowledge in auditory perceptual filling-in, as it combines elements of auditory scene analysis and the extraction of speech, as well as the perception of lower-level musical features of timbre characterizing human vocalizations. The MIDI vocals illusion, first described in a YouTube video in 2015 by user MonotoneTim (Auditory Illusions: Hearing Lyrics Where There Are None), captures a peculiar effect, wherein familiar songs converted to a format consisting exclusively of digital piano notes still evoke the perception of the vocal elements present in the original audio, albeit somewhat muffled (MonotoneTim, 2015). These converted audio files are in the MIDI format, which relies on frequency analysis of complex waveforms from .wav, .mp3, or other such digital audio files to approximate the original sound using a set of digital notes at each time point (Brown and Puckette, 1992; Forberg, 1998). While this method of audio conversion is typically used to create a digitized score of the music, the notes produced can be played with a digital instrument of the user’s choice, meaning that all percussion, vocal, and instrumental elements are represented by, in many cases, digital piano notes.

Anecdotal accounts of the MIDI vocals illusion suggest distinct perceptual qualities that are driven by prior exposure to the original song, which would, in this case, represent a veridical source prediction (Bharucha and Stoeckig, 1987). Specifically, listeners indicate a richer perceptual experience of apparent human vocal elements in MIDI-converted songs if they have heard the original audio. This form of prior knowledge, rooted in the long-term and frequent exposure to the original song, is analogous to what is captured by word stimuli in the context of continuity illusion (Shahin et al., 2009). It also resembles the enhanced ability to parse speech in noise-vocoded audio (Hervais-Adelman et al., 2011) and the detection of higher-order violations of musical expectations by trained listeners (Fujioka et al., 2004). At the same time, statistical regularities in the sensory signal allowing listeners to identify human vocals as the source may be conserved during the MIDI conversion process. In this study, we investigate the perceptual quality of human vocal elements perceived in MIDI-converted audio through two experiments: one asking listeners to report non-specific vocal elements (i.e., the sound of voices) and another asking listeners to report the perception of words (i.e., a more precise percept). By presenting MIDI-converted audio corresponding to songs that have never been heard by the listener and progressively exposing them to the original excerpts of a subset of those stimuli, the timeline over which prior knowledge can begin influencing perception, and the qualities of this influence, can be characterized. We predict that the perception of words will be more likely to occur when the listener has heard original intact excerpts. In addition, if statistical regularities inherent to a vocal stimulus are preserved during the MIDI conversion process, we expect listeners to perceive non-specific vocals to a greater degree only when listening to MIDI-converted sounds corresponding to an original excerpt containing vocals and not exclusively instrumental elements. This low-precision percept, which we hypothesize relies on information in the ongoing stimulus, should then occur regardless of prior exposure to the original excerpts.

## 2 Materials and methods

### 2.1 Participants

A total of 39 participants (28 female and 11 male individuals) were recruited to take part in one of two experiments, asking them to report the incidence and duration of perceiving specific vocal elements in the presented audio clips. For the first group, the listeners reported the incidence of vocal elements generally, while the second group was instructed to report the incidence of discernible words within the presented audio. Following the exclusion of participants who reported prior exposure to any of the included songs, data from 19 participants in the non-specific vocal element group [13 female individuals, 17 right-handed, mean age of 24.7 ± 8.6 year (sd)] and 14 participants in the discernible words group [10 female individuals, 12 right-handed, mean age of 20.1 ± 1.7 years (sd)] were submitted for final analysis. The participants were recruited from the local community, as well as through extra-credit-eligible course enrollments within the Bradley University Department of Psychology. The inclusion criteria were as follows: age between 18 and 45 years, no diagnosis of any form of hearing impairment during their lifetime, and no history of neurological conditions or seizures. Written informed consent was acquired from each participant in accordance with the ethical guidelines set forth by the Committee on the Use of Human Subjects in Research (CUHSR) and the Internal Review Board (IRB) of Bradley University. The participants were compensated at a rate of $10 per half hour, rounded up to the next 15-min increment.

### 2.2 Procedure

**Figure 1:**
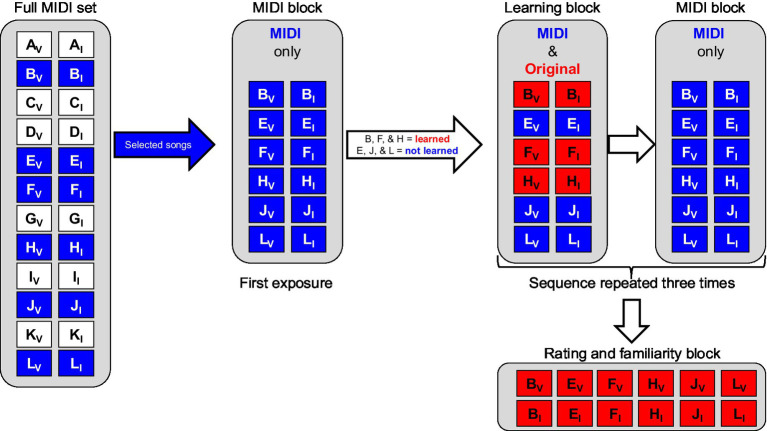
Session structure. For each participant, 6 of the 12 possible songs (A-L) were randomly selected, with the chosen songs and subsequent exposure condition assignments being counterbalanced across the participants. The participants first completed a MIDI-only listening task, in which they were presented with MIDI-converted excerpts of songs (depicted in blue) containing either instrumental elements alone (denoted with subscript I) or both instrumental and vocal elements (denoted with subscript V). They were asked to indicate the incidence of the perception of vocal elements by pressing and holding the spacebar. For experiment 1, the listeners reported non-specific vocal elements, while for experiment 2, the participants reported the perception of words. Following an initial MIDI-only block, the participants completed a sequence consisting of a learning block followed by a MIDI-only block three times. For the learning block, instrumental- and vocal-containing excerpts were presented in their original, unconverted format (depicted in red). The remaining three songs were only presented in their MIDI-converted format. Following three iterations of this sequence, the participants completed a rating and familiarity task, in which they listened to the original excerpts of all six of their assigned songs and indicated the appeal of those excerpts on a scale from one (highly unappealing) to four (highly appealing), as well as whether or not they had ever heard the song prior to their participation in the study.

The participants completed a single session (Figure 1) consisting of a music listening task divided into alternating MIDI-only blocks and learning blocks. For both the MIDI-only and learning blocks, the participants were asked to press and hold the spacebar whenever they perceived a vocal element in the sound stimulus, as specified by their instructions. Specifically, one group was instructed to press and hold the spacebar whenever they perceived the presence of vocal elements of any kind, while the second group was instructed to press and hold the spacebar when they perceived the presence of words in the presented audio. Following the completion of these blocks, a single run of a song familiarity and appeal rating task was completed. For this task, the participants were presented with the original version of all of their assigned song excerpts and asked to indicate the appeal of the song on a scale from one to four, as well as to indicate whether they had ever heard the song prior to their arrival at the laboratory that day. If a participant indicated any prior familiarity with any of their assigned songs, their data were excluded from the analysis.

#### 2.2.1 Stimuli and tasks

All behavioral tasks were prepared and implemented using the Presentation stimulus delivery software (Neurobehavioral Systems, Albany, CA; stimuli and codes are available on the Open Science Framework preprint platform at https://osf.io/m96ya/orosf.io/m96ya/). Visual prompts were presented on a 24-inch screen with a resolution of 1,440 by 900 pixels, a viewing distance of 60 cm, and a refresh rate of 60 Hz. MIDI and original song excerpts were presented as uncompressed .wav files with a resolution of 16-bit/44100 Hz. These stimuli were delivered through over-ear, wired (3.5 mm audio jack), noise-canceling JAM Audio model HX-HP303 headphones (JAM USA, Commerce Township, MI), with a frequency range of 20 Hz–20 kHz. Before beginning the task, each participant adjusted the headphone volume to ensure the perceptibility of presented sounds and to prevent discomfort due to excessive loudness. All tasks were completed in a closed, sound-attenuated behavioral run room, without the use of active noise canceling (ANC) features in the headphones.

#### 2.2.2 Audio stimulus processing and production

**Figure 2:**
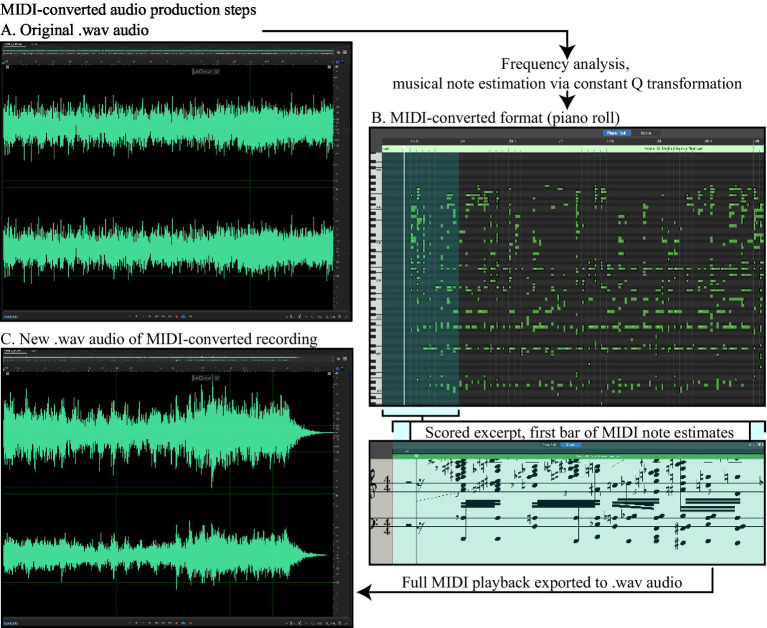
Audio file formats and their conversion to MIDI. Original audio was loaded first as an MP3 file into Adobe Audition, where it was exported to .wav format with 16-bit/44100 Hz resolution. An example of the original waveform is depicted in **(A)**, with the Y-axis corresponding to amplitude in decibels (dB). MIDI conversion, based on frequency analysis applied to each sample point, yielded a “piano roll” of digital notes, the corresponding output of which is depicted along with a scored version in **(B)**. The MIDI playback using the Parallel Earth Piano voice was then re-recorded as a .wav file with settings identical to those of the original .wav file [corresponding waveform shown in **(C)**].

For each of the 12 songs in the full set (see Supplementary materials S1 for the full list), we extracted two 10-s excerpts: one containing an uninterrupted stream of vocals accompanying the instrumental elements and the other containing only instrumental elements. Every excerpt was then converted into the MIDI format by processing extracted .wav files using a constant Q transform (Brown, 1991; Brown and Puckette, 1992). This process converted the original waveform audio into a digital “piano roll” of discrete digital piano notes representing an estimate of the frequency content of the complex waveform at each time point, all played in the same digital piano “voice” (Figure 2B). This process resulted in a stimulus set of 48 audio clips: 12 originals with vocals, 12 originals without vocals, and their respective MIDI-format versions. The MIDI-format clips were then loaded into Garage Band (version 10.4.8, Apple, Inc., Cupertino, CA) using the Parallel Earth Piano voice, with a noise setting of five; tremolo, chorus, delay time, and delay mix all set to zero; and a reverberation level of 10 (Figure 2A shows the original waveform and the corresponding MIDI-converted excerpt (B) from an example track). These settings were selected during behavioral piloting, in which the authors listened to the MIDI-converted audio corresponding to a song known to have induced the perception of vocal elements in prior demonstrations (MonotoneTim, 2015). The MIDI playback was then exported to .wav format with audio settings identical to the original samples using Adobe Audition (Build 24.2.0.83, Adobe, Inc., San Jose, CA Figure 2C). All songs were selected and utilized in accordance with Fair Use provisions set out by the US Copyright Act § 107 (1976).

#### 2.2.3 Music listening tasks

For each experiment, the session was divided into eight task blocks, alternating between two types: a MIDI-only block and a learning block, wherein a random, pre-selected subset of the participant’s songs was presented in their original format, along with the MIDI-only versions of the remaining songs. For the low-precision illusion experiment, the participants were instructed to press and hold the spacebar while listening to the stimuli when they perceived vocal elements of any kind in the audio. For the high-precision illusion experiment, the listeners were instructed to press and hold the spacebar any time they perceived the presence of words in the audio. Apart from this difference in instructions, task and stimulus parameters were identical across the experiments. Each block consisted of 24 trials, presented in random order. For the MIDI-only block, two trials of each 10-s MIDI-converted audio file were presented. These MIDI-converted audio clips corresponded to the six songs assigned to the participant and included two trials of each song’s vocals-present and vocals-absent excerpts. Following this, a learning block was completed with identical task instructions. For this block, three of the assigned songs were presented in their original unconverted format, while the remaining tracks were presented in the MIDI-converted format only. These MIDI-only and learning blocks alternated such that, by each new MIDI-only block, the participant had been exposed to the original versions of three of their assigned songs twice, while only ever hearing the MIDI versions of the remaining three. A total of three learning blocks and four MIDI-only blocks were completed, yielding a four-level factor of phase for the MIDI-only runs (i.e., before exposure to the original versions, and after one, two, and three exposures). Following this, the participants were presented with all 12 original versions of their assigned song excerpts and were asked to indicate whether they had ever heard the audio prior to the experimental session. They were then asked to rate the appeal of the song on a scale from one to four, with one corresponding to a rating of highly unappealing and four corresponding to a rating of highly appealing. If any of the excerpts played during this final task were identified as previously heard by the participant, that participant’s data were excluded from any further analysis. This exclusion criterion enabled us to effectively isolate the effect of *de novo* exposure to original excerpts on the emergence of the perception of vocal elements within the MIDI audio files.

### 2.3 Data analysis

For each group, the incidence of the MIDI vocals illusion was measured as the mean total duration (in seconds) of spacebar presses for each trial type during the MIDI-only blocks (two exposures to each excerpt yielded a maximum of 20 s for each excerpt type). Although we targeted excerpts with maximally uninterrupted vocal elements for our vocals-present stimuli, we accounted for brief (< 1-s duration) vocal pauses by allowing multiple button presses and calculating the total duration across these presses for each trial. This measure was then submitted to a 4 by 2 by 2 repeated-measures ANOVA (JASP, version 0.16.3, JASP Team, Amsterdam, Netherlands), with the factors of prior learning blocks completed (none, one, two, or three exposures to the original versions of “learned” songs), exposure condition (MIDI audio corresponding to those presented in either their original format or MIDI-only version during the learning blocks), and vocal presence (MIDI audio corresponding to excerpts originally containing vocal elements or only instrumental elements). The inclusion of non-vocal excerpts allowed us to probe the presence of false positives (i.e., perceiving vocals in a converted excerpt whose original file contains none). All reported statistical values were Greenhouse–Geisser corrected for violations of sphericity for the learning blocks factor, and all post-hoc comparison *p*-values were adjusted using the Bonferroni correction for multiple comparisons.

## 3 Results

### 3.1 Experiment 1: low-precision illusion

**Figure 3:**
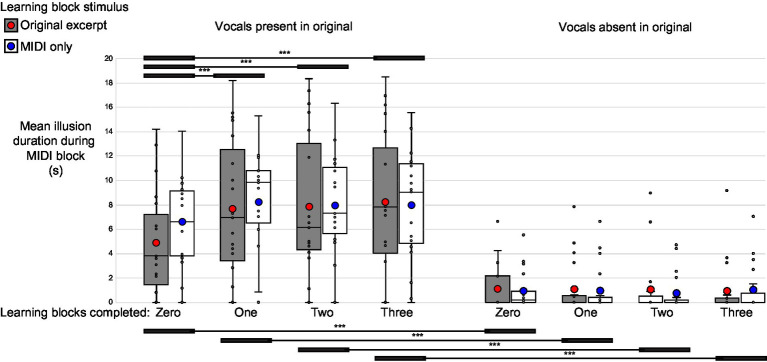
Low-precision illusion. Mean duration of perceived non-specific vocal elements during the MIDI-only blocks. The left set of eight bars corresponds to MIDI stimuli converted from original excerpts containing vocals, while the right set of eight bars corresponds to MIDI stimuli derived from instrumental-only excerpts. Gray bars depict illusion durations for MIDI stimuli whose corresponding original excerpts were presented during the learning blocks, while white bars depict illusion durations for MIDI stimuli only ever presented in the MIDI format, even during the learning blocks. The mean is shown as a large circle (red for MIDIs corresponding to learned original excerpts, and blue for MIDIs corresponding to never-heard originals), the median as a horizontal black line, and individual data points as small circles. The box itself captures the interquartile range (top being the 75th percentile, and bottom being the 25th percentile). The mean MIDI vocals illusion duration showed a significant phase by vocal presence interaction, driven by the rapid emergence of the illusion over time, with MIDI blocks two, three, and four yielding illusion durations for MIDI stimuli corresponding to originals with vocal elements that were significantly greater than the duration observed during the first MIDI block. The MIDI illusion duration was significantly greater for stimuli corresponding to original excerpts containing vocals than for those containing none, even during the first block (i.e., prior to any exposure during the learning block). No apparent effect of exposure to the original excerpts during the learning blocks was observed. *p < 0.05, **p < 0.01, ***p < 0.001.

Among the group reporting the perception of non-specific vocal elements during the MIDI blocks, 18 of the 19 participants (95%) experienced the perception of vocals in response to MIDI-converted excerpts containing vocals, while 10 (53%) experienced the perception of vocals for a total duration exceeding 1 s in response to MIDI-converted instrumental-only excerpts. The mean total duration of the MIDI vocals illusion, submitted to a phase by learning by vocal presence repeated-measures ANOVA, revealed significant main effects of phase (F_3,54_ = 5.64, *p* = 0.01, η_p_^2^ = 0.24) and vocal presence (F_1,18_ = 63.98, *p* < 0.001, η_p_^2^ = 0.78), with the duration of the illusion being greater for the second, third, and fourth MIDI blocks compared to the first and significantly longer for MIDI stimuli corresponding to original excerpts containing vocals than for those only containing instrumental elements. In addition, a significant phase by vocal presence interaction was observed (F_3,54_ = 9.21, *p* < 0.001, η_p_^2^ = 0.34). Post-hoc comparisons using Bonferroni correction revealed that this was driven by a progressive increase in the mean total duration of the MIDI vocals illusion only for those MIDI files corresponding to original excerpts containing vocal elements, and they suggested no credible evidence for a significant change in the duration of the MIDI vocals illusion for MIDI files corresponding to original excerpts containing only instrumental elements (Figure 3, Supplementary material S2; Supplementary Table 4).

### 3.2 Experiment 2: high-precision illusion

**Figure 4:**
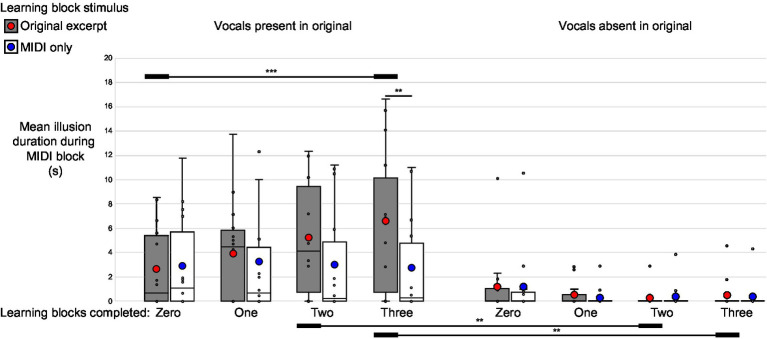
High-precision illusion. Phase by learning by vocal presence interaction. For the listeners instructed to report the presence of words during their session, the MIDI illusion only emerged for MIDI excerpts originally containing vocals and corresponding to original songs to which listeners were exposed during the learning blocks. This is evident in a significant increase in the reported duration of word presence during the final block of MIDI trials compared to the first (i.e., prior to exposure to the original excerpts), as well as in a significantly greater duration of word perception for MIDI excerpts corresponding to learned songs in the final and penultimate MIDI blocks. *p < 0.05, **p < 0.01, ***p < 0.001.

In the group reporting the perception of words during the MIDI blocks, 10 listeners (79%) experienced the perception of words in response to MIDI-converted excerpts containing vocals, while six (43%) experienced the perception of words for a total duration exceeding 1 s in response to MIDI-converted instrumental-only excerpts. The mean total duration of reported perception of the MIDI vocals illusion among the participants who were asked to report the incidence of words during the MIDI block, when submitted to the same analysis as described above, revealed a main effect of vocal presence in the corresponding original excerpts (F_1,13_ = 11.63, *p* = 0.005, η_p_^2^ = 0.47), with the mean duration of perceived words during the MIDI blocks being significantly greater for stimuli corresponding to vocal-containing excerpts than for those containing only instrumental elements. A significant phase by exposure interaction (F_3,39_ = 5.92, *p* = 0.013, η_p_^2^ = 0.31) was driven by the progressive increase in the illusion duration for MIDI stimuli corresponding to original excerpts to which the listeners were exposed during the learning block, with repeated exposures to the originals. Finally, a phase by exposure by vocal presence interaction (F_3,39_ = 7.22, *p* = 0.005, η_p_^2^ = 0.36) was observed. Post-hoc comparisons using Bonferroni correction showed that this was driven by the progressive emergence of the MIDI vocals illusion only for excerpts corresponding to original versions containing vocals and to which the listener had been exposed during the learning blocks (Figure 4; Supplementary material S3; Supplementary Table 4).

## 4 Discussion

### 4.1 The MIDI vocals illusion

The MIDI vocals illusion is an as-yet unstudied auditory perceptual phenomenon that provides a unique opportunity to examine the role of prior knowledge in the construction of perception in the face of degraded sensory evidence. Although anecdotal accounts of the illusion describe the perception of lyrics in songs converted exclusively to a digital piano format, they only refer to highly popular songs, and it has not yet been possible to characterize the effect of prior knowledge on the quality of the illusion (MonotoneTim, 2015). The present study addresses this by observing the illusion as it occurs in response to never-before-heard songs and operationalizes prior information by exposing each listener to the original version of half of their assigned songs over the course of the session. The specific illusion quality targeted by the present study is precision, operationalized via instructions to report the occurrence of either non-specific vocal elements of any kind (less precise) or words of any kind (more precise).

### 4.2 Imprecise perception of vocals and online statistical learning

The occurrence of the MIDI vocals illusion in the low-precision group reporting the perception of non-specific vocal elements is not, by itself, surprising because the process of MIDI conversion can be considered a form of spectral degradation not unlike the vocoding of speech (Roberts et al., 2011; Souza and Rosen, 2009). Two notable patterns in the current findings suggest that, for the low-precision group, non-specific vocal elements are extracted through online learning of statistical regularities in the ongoing sensory stream, in accordance with mechanisms of predictive coding serving the perception of speech and music (Narmour, 1991; Bregman, 1994; Rohrmeier and Koelsch, 2012). Specifically, the listeners reliably perceived vocal elements in the MIDI-converted files containing vocals, with false positives being comparatively rare for the instrumental-only excerpts. This sensitivity to the presence of converted vocals, although nonspecific, supports the well-established sensitivity of the human acoustical perceptual system to sounds originating from the human vocal apparatus (Belin et al., 2000; Binder et al., 2000). It appears here that essential features supporting source identification are preserved in the spectral content of the MIDI-converted stimulus (Latinus and Belin, 2011). In addition, prior exposure to the original excerpts did not affect the emergence of this low-precision illusion, suggesting that the identification of a human voice embedded in a MIDI-converted sound does not require prior exposure, bolstering the sufficiency of bottom-up signals. In this way, the low-precision form of the MIDI vocals illusion can be considered analogous to the bottom-up influences that produce the continuity illusion (Samuel, 1981); the extraction of human speech from vocoded speech stimuli (Shannon et al., 1995; Souza and Rosen, 2009); and the perception of tone, key, and timbre in music (Pearce and Wiggins, 2006; Rohrmeier and Koelsch, 2012).

### 4.3 Word perception and prior exposure

For the group reporting the incidence of words (high precision), prior exposure to original excerpts was essential for the emergence of the illusion. Within the framework of predictive coding, prior exposure provides a veridical source prediction (Bharucha and Stoeckig, 1987), which serves top-down perceptual filling-in. For speech extraction from degraded signals or noise-vocoded speech, this effect is analogous to the transfer of discrimination performance following exposure to previously heard audio to a vocoded speech sample constructed from a different set of frequency bands (Hervais-Adelman et al., 2011). In the case of music perception, chord priming effects (Bharucha and Stoeckig, 1987; Bigand and Pineau, 1997), or the effects of long-term exposure through music listening or training (Kern et al., 2022; Fujioka et al., 2004), represent top-down expectations informed by experience, analogous to the current study’s presentation of original excerpts and its effects on the sophistication of perceived vocals.

### 4.4 Future directions

Given the present results, potential future studies of the MIDI vocals illusion can provide further insight into the distinct contributions of different types of expectations in the construction of auditory perception. For example, while the neural bases of tinnitus, musical hallucinations, and auditory hallucinations associated with psychosis have been characterized within the predictive coding framework (Kumar et al., 2014; Henry et al., 2014; Horga et al., 2014), it would be of interest to observe, in participants listening to physically identical MIDI-converted stimuli, whether prior exposure to original excerpts leads to measurable changes in brain activity during MIDI listening. Specifically, the current experimental procedure allows for the rapid onset of high-precision illusory perceptions based on prior learning and, with the stimuli of interest being the same MIDI-converted excerpts throughout the study, provides strong control of the physical properties of the stimuli.

The recovery of words from MIDI-converted audio following exposure to the intact original sound can also motivate future studies investigating the strength of this top-down expectation, both within the auditory domain and cross-modally. For example, by converting popular songs to MIDI format after substituting new, tempo-matched vocals for the originals, whether or not a naive listener continues to perceive the original vocals in the MIDI-converted file could establish the overriding strength of this filling-in, as it would be contradictory to the true physical nature of the original sound. Top-down cross-modal influences are also of interest. For example, during the McGurk effect, visual cues depicting mouth movements associated with different formants distort a uniform auditory phoneme (Green and Gerdman, 1995), and it may be the case that visually presented subtitles accompanying a MIDI-converted speech stimulus bias the perception of the sound toward the visual cue, an effect that otherwise facilitates second-language parsing (Vulchanova et al., 2015).

### 4.5 Limitations

While the current findings demonstrate a unique role of prior exposure in determining the perceptual quality of a novel illusion, there are several limitations to consider. In the case of stimulus delivery methods, the auditory setup in the current study is less than ideal. In the future, and in accordance with audiological standards, it will be necessary to utilize high-quality headphones with a well-established frequency response curve that minimizes signal degradation or spectral distortions (Poldy, 2012). In terms of our participant sample, our screening procedure relied on self-reported hearing impairment for exclusion. While it is unlikely that a significant portion of the young adult population we sampled has undiagnosed hearing impairment (Hoffman et al., 2017), it is estimated that approximately 13% of Americans aged 12 and older experience some form of hearing loss (Lin et al., 2011). A more comprehensive hearing test prior to inclusion in similar studies will ensure comparable sensitivity and acuity in future samples. Finally, to better characterize the perceptual quality of the MIDI vocals illusion, it will be helpful to incorporate a behavioral task yielding quantifiable measures of precision. For example, d-prime measures for simple detection of vocal elements, or speech discriminability in a two-alternative forced choice task, would provide useful metrics that consider response biases and possible false alarms (Macmillan, 2002).

## 5 Conclusion

Perceptual filling-in of gaps inherent to sensory evidence is a core feature of perception and has been extensively studied through the lens of illusions. In this first investigation of the MIDI vocals illusion, the precision of the percept, and the influence of prior knowldge rooted in exposure to original excerpts, were of central interest. Here, the emergence of the imprecise perception of missing vocals relies on bottom-up sensory processing, while the emergence of the illusory perception of words, constituting a more precise illusion, relies on a top-down expectation rooted in prior knowledge gained through exposure to the original excerpt.

## Data Availability

Stimuli, data, task and analysis scripts, and supplementary materials can be found on the Open Science Framework repository at DOI: https://osf.io/m96ya/ as well as in the article/Supplementary material.
