# Supplementary material for: The impact of prior knowledge on perceiving vocal elements in MIDI-converted music
Source: Front Psychol. 2025 Sep 30;16:1565292. doi: 10.3389/fpsyg.2025.1565292 (PMC12518094; doi:10.3389/fpsyg.2025.1565292)
Supplement: Supplementary file 1 [file Supplementary_file_1.docx]

Songs used

1. Olympics. (1966). I’ll do a little bit more. On *Something Old, Something New.* Ace Records
2. Pink Floyd. (1987). *Learning to Fly.* On *A Momentary Lapse of Reason.* Pink Floyd Ltd.; Sony Music Entertainment.
3. Secret Garden. (2019). Beautiful feat. Brian Kennedy. On *Storyteller*. Universal Music A/S.
4. Sex Pistols. (1977). Holidays In the Sun. On *Never Mind the Bollocks, Here’s the Sex Pistols*. Warner Records, Inc.
5. Isak Danielson. (2021). If You Ever Forget That You Love Me. On *Tomorrow Never Came.* IOD Entertainment.
6. Ingrid Michaelson. (2009). Everybody. On *Everybody*. Spirit Music Group; Universal Music Enterprises, UMG Recordings, Inc.
7. Gandalf. (1968). Golden Earrings. On *Gandalf*. Capitol Records, LLC.
8. DJ Shadow feat. Chris James. (2006). You Made It. On *The Outsider*. A&M (UK) Ltd.
9. Wax. (1995). California. On *13 Unlucky Numbers.* Interscope Records.
10. Olga Pasichnyk and Natalya Pasichnyk. (2010). 19 Polish Songs, Op. 74: No. 1. Zyczenie. On *Chopin: Songs.* Naxos.
11. Cody Fry. (2021). Photograph. On *Pictures of Mountains.* Cody Fry Music, LLC; UMG Recordings, Inc.
12. Danny Davis and The Nashville Brass. (1967). Go Speed Racer Go (comp. Nobuyoshi Koshibe). On *Speed Racer Classic Original Theme Song.* Speed Classic Music (BMI).
