# Supplementary material for: The impact of prior knowledge on perceiving vocal elements in MIDI-converted music
Source: Front Psychol. 2025 Sep 30;16:1565292. doi: 10.3389/fpsyg.2025.1565292 (PMC12518094; doi:10.3389/fpsyg.2025.1565292)
Supplement: Supplementary file 3 [file Supplementary_file_3.docx]

**Experiment 2: Perception of words (high-precision illusion)**

Mean total duration (seconds) word perception during MIDI-only blocks submitted to 4 x 2 x 2 repeated-measures ANOVA

**Learning blocks completed** (None, 1, 2, or 3) by **Exposure** (original excerpts or MIDI-only excerpts in learning blocks) by **Vocal presence** in original excerpt (vocals present or instrumental only)

| **Learning blocks** | | **Exposure** | | **Vocal presence in original** | **Mean** | | | **SD** | |  |
| --- | --- | --- | --- | --- | --- | --- | --- | --- | --- | --- |
| None |  | Original |  | Instrumental only |  | 1.152 |  | 2.680 |  |  |
|  |  |  |  | Vocals |  | 2.268 |  | 3.364 |  |  |
|  |  | MIDI only |  | Instrumental only |  | 1.162 |  | 2.809 |  |  |
|  |  |  |  | Vocals |  | 2.878 |  | 3.967 |  |  |
| 1 |  | Original |  | Instrumental only |  | 0.506 |  | 0.981 |  |  |
|  |  |  |  | Vocals |  | 3.934 |  | 4.229 |  |  |
|  |  | MIDI only |  | Instrumental only |  | 0.268 |  | 0.783 |  |  |
|  |  |  |  | Vocals |  | 3.240 |  | 4.749 |  |  |
| 2 |  | Original |  | Instrumental only |  | 0.240 |  | 0.766 |  |  |
|  |  |  |  | Vocals |  | 5.246 |  | 4.732 |  |  |
|  |  | MIDI only |  | Instrumental only |  | 0.346 |  | 1.026 |  |  |
|  |  |  |  | Vocals |  | 3.005 |  | 4.542 |  |  |
| 3 |  | Original |  | Instrumental only |  | 0.482 |  | 1.266 |  |  |
|  |  |  |  | Vocals |  | 6.614 |  | 5.910 |  |  |
|  |  | MIDI only |  | Instrumental only |  | 0.341 |  | 1.152 |  |  |
|  |  |  |  | Vocals |  | 2.735 |  | 4.062 |  |  |
|  | | | | | | | | | | |

**Table 1. Word perception duration, Experiment 2.** Mean total duration of vocals perception during MIDI blocks and standard deviation are shown. Means are calculated for every condition, according to the number of learning blocks completed, whether the listener hears corresponding original excerpts or only MIDI versions of each file during learning blocks, and whether the MIDI stimulus corresponds to an original excerpt containing vocal elements or instrumental elements only.

| Main effects | df_num | df_denom | F | **η²_p_** | *p* |
| --- | --- | --- | --- | --- | --- |
| Learning blocks | 3 | 39 | 0.45 | .03 | .56 |
| Exposure | 1 | 13 | 2.70 | .17 | .13 |
| Vocal presence | 1 | 13 | 11.63 | .47 | .005 |
| Interactions | df_num | df_denom | F | **η²_p_** | *p* |
| Learning blocks x Exposure | 3 | 39 | 5.92 | .31 | .013 |
| Learning blocks x Vocal presence | 3 | 39 | 7.90 | .38 | .005 |
| Exposure x Vocal presence | 1 | 13 | 3.08 | .19 | .103 |
| Learning blocks x Exposure x Vocal presence | 3 | 39 | 7.23 | .36 | .005 |

**Table 2. rANOVA omnibus test results, Experiment 2**. Mean total durations of word perception duration during MIDI blocks were submitted to a repeated-measures ANOVA on the factors of prior learning blocks completed (0, 1, 2, or three), learning block exposure (original or MIDI only), and vocal presence in corresponding original excerpts (vocals present or instrumental only). Tests of Sphericity (provided below) indicated a violation of this assumption for the prior learning blocks factor, and all *p*-values associated with this factor have been Greenhouse-Geisser corrected.

| Effects | Mauchly's W | df | *p* |
| --- | --- | --- | --- |
| Learning blocks | 0.058 | 5 | <.001 |
| Learning blocks x Exposure | 0.182 | 5 | 0.001 |
| Learning blocks x Vocal presence | 0.164 | 5 | <.001 |
| Learning blocks x Exposure x Vocal presence | 0.343 | 5 | 0.029 |

**Table 3. Test of Sphericity, Experiment 2**. Mauchly's Test of Sphericity, conducted on the factor of prior learning blocks completed (None, 1, 2, or 3), revealed a violation of this assumption for its main effect, its interactions with the factors of vocal presence and exposure conditions, as well as the three-way learning block by exposure condition by vocal presence interaction.

| **Learning blocks completed (0/1/2/3)**  **Exposure (O/M)**  **Vocal presence (Y/N)** | |  |  |  |  |
| --- | --- | --- | --- | --- | --- |
|  |  | **Mean** |  |  |  |
|  |  | **difference** | **t(13)** | **Cohen's d** | ***p*_Bonf_** |
| None, O, Y | 1, O, Y | -1.306 | -1.776 | -0.386 | 1.000 |
|  | 2, O, Y | -2.618 | -3.560 | -0.774 | 0.070 |
|  | 3, O, Y | -3.985 | -5.419 | -1.178 | <.001*** |
|  | None, M, Y | -0.250 | -0.307 | -0.074 | 1.000 |
|  | 1, M, Y | -0.612 | -0.628 | -0.181 | 1.000 |
|  | 2, M, Y | -0.376 | -0.386 | -0.111 | 1.000 |
|  | 3, M, Y | -0.106 | -0.109 | -0.031 | 1.000 |
|  | None, O, N | 1.476 | 1.290 | 0.436 | 1.000 |
|  | 1, O, N | 2.122 | 1.697 | 0.627 | 1.000 |
|  | 2, O, N | 2.388 | 1.909 | 0.706 | 1.000 |
|  | 3, O, N | 2.146 | 1.716 | 0.634 | 1.000 |
|  | None, M, N | 1.466 | 1.240 | 0.433 | 1.000 |
|  | 1, M, N | 2.360 | 1.851 | 0.698 | 1.000 |
|  | 2, M, N | 2.282 | 1.790 | 0.674 | 1.000 |
|  | 3, M, N | 2.287 | 1.794 | 0.676 | 1.000 |
| 1, O, Y | 2, O, Y | -1.312 | -1.784 | -0.388 | 1.000 |
|  | 3, O, Y | -2.679 | -3.643 | -0.792 | 0.053 |
|  | None, M, Y | 1.056 | 1.084 | 0.312 | 1.000 |
|  | 1, M, Y | 0.694 | 0.853 | 0.205 | 1.000 |
|  | 2, M, Y | 0.930 | 0.954 | 0.275 | 1.000 |
|  | 3, M, Y | 1.200 | 1.231 | 0.355 | 1.000 |
|  | None, O, N | 2.782 | 2.225 | 0.822 | 1.000 |
|  | 1, O, N | 3.428 | 2.996 | 1.013 | 0.708 |
|  | 2, O, N | 3.694 | 2.954 | 1.092 | 0.658 |
|  | 3, O, N | 3.452 | 2.760 | 1.020 | 1.000 |
|  | None, M, N | 2.772 | 2.174 | 0.819 | 1.000 |
|  | 1, M, N | 3.666 | 3.100 | 1.084 | 0.515 |
|  | 2, M, N | 3.588 | 2.814 | 1.060 | 0.926 |
|  | 3, M, N | 3.593 | 2.818 | 1.062 | 0.917 |
| 2, O, Y | 3, O, Y | -1.367 | -1.859 | -0.404 | 1.000 |
|  | None, M, Y | 2.368 | 2.430 | 0.700 | 1.000 |
|  | 1, M, Y | 2.006 | 2.059 | 0.593 | 1.000 |
|  | 2, M, Y | 2.242 | 2.754 | 0.663 | 0.998 |
|  | 3, M, Y | 2.512 | 2.578 | 0.742 | 1.000 |
|  | None, O, N | 4.094 | 3.274 | 1.210 | 0.280 |
|  | 1, O, N | 4.740 | 3.790 | 1.401 | 0.066 |
|  | 2, O, N | 5.006 | 4.374 | 1.480 | 0.021* |
|  | 3, O, N | 4.764 | 3.809 | 1.408 | 0.062 |
|  | None, M, N | 4.084 | 3.203 | 1.207 | 0.331 |
|  | 1, M, N | 4.978 | 3.905 | 1.471 | 0.045* |
|  | 2, M, N | 4.900 | 4.143 | 1.448 | 0.033* |
|  | 3, M, N | 4.905 | 3.847 | 1.450 | 0.053 |
| 3, O, Y | None, M, Y | 3.735 | 3.834 | 1.104 | 0.031* |
|  | 1, M, Y | 3.373 | 3.463 | 0.997 | 0.105 |
|  | 2, M, Y | 3.609 | 3.705 | 1.067 | 0.048* |
|  | 3, M, Y | 3.879 | 4.765 | 1.146 | 0.002** |
|  | None, O, N | 5.462 | 4.367 | 1.614 | 0.012* |
|  | 1, O, N | 6.108 | 4.884 | 1.805 | 0.003** |
|  | 2, O, N | 6.373 | 5.096 | 1.884 | 0.001** |
|  | 3, O, N | 6.131 | 5.358 | 1.812 | 0.002** |
|  | None, M, N | 5.451 | 4.276 | 1.611 | 0.015* |
|  | 1, M, N | 6.346 | 4.977 | 1.876 | 0.002** |
|  | 2, M, N | 6.267 | 4.916 | 1.852 | 0.002** |
|  | 3, M, N | 6.272 | 5.304 | 1.854 | 0.001** |
| None, M, Y | 1, M, Y | -0.362 | -0.492 | -0.107 | 1.000 |
|  | 2, M, Y | -0.126 | -0.171 | -0.037 | 1.000 |
|  | 3, M, Y | 0.144 | 0.196 | 0.043 | 1.000 |
|  | None, O, N | 1.727 | 1.460 | 0.510 | 1.000 |
|  | 1, O, N | 2.373 | 1.861 | 0.701 | 1.000 |
|  | 2, O, N | 2.638 | 2.069 | 0.780 | 1.000 |
|  | 3, O, N | 2.396 | 1.879 | 0.708 | 1.000 |
|  | None, M, N | 1.716 | 1.500 | 0.507 | 1.000 |
|  | 1, M, N | 2.610 | 2.087 | 0.772 | 1.000 |
|  | 2, M, N | 2.532 | 2.025 | 0.748 | 1.000 |
|  | 3, M, N | 2.537 | 2.029 | 0.750 | 1.000 |
| 1, M, Y | 2, M, Y | 0.236 | 0.320 | 0.070 | 1.000 |
|  | 3, M, Y | 0.506 | 0.687 | 0.149 | 1.000 |
|  | None, O, N | 2.088 | 1.638 | 0.617 | 1.000 |
|  | 1, O, N | 2.734 | 2.312 | 0.808 | 1.000 |
|  | 2, O, N | 3.000 | 2.353 | 0.887 | 1.000 |
|  | 3, O, N | 2.758 | 2.163 | 0.815 | 1.000 |
|  | None, M, N | 2.078 | 1.661 | 0.614 | 1.000 |
|  | 1, M, N | 2.972 | 2.597 | 0.878 | 1.000 |
|  | 2, M, N | 2.894 | 2.314 | 0.855 | 1.000 |
|  | 3, M, N | 2.899 | 2.318 | 0.857 | 1.000 |
| 2, M, Y | 3, M, Y | 0.270 | 0.367 | 0.080 | 1.000 |
|  | None, O, N | 1.853 | 1.453 | 0.548 | 1.000 |
|  | 1, O, N | 2.499 | 1.960 | 0.739 | 1.000 |
|  | 2, O, N | 2.764 | 2.337 | 0.817 | 1.000 |
|  | 3, O, N | 2.522 | 1.978 | 0.745 | 1.000 |
|  | None, M, N | 1.842 | 1.473 | 0.545 | 1.000 |
|  | 1, M, N | 2.737 | 2.188 | 0.809 | 1.000 |
|  | 2, M, N | 2.658 | 2.323 | 0.786 | 1.000 |
|  | 3, M, N | 2.663 | 2.129 | 0.787 | 1.000 |
| 3, M, Y | None, O, N | 1.583 | 1.241 | 0.468 | 1.000 |
|  | 1, O, N | 2.229 | 1.748 | 0.659 | 1.000 |
|  | 2, O, N | 2.494 | 1.956 | 0.737 | 1.000 |
|  | 3, O, N | 2.252 | 1.904 | 0.666 | 1.000 |
|  | None, M, N | 1.572 | 1.257 | 0.465 | 1.000 |
|  | 1, M, N | 2.467 | 1.972 | 0.729 | 1.000 |
|  | 2, M, N | 2.388 | 1.910 | 0.706 | 1.000 |
|  | 3, M, N | 2.393 | 2.091 | 0.707 | 1.000 |
| None, O, N | 1, O, N | 0.646 | 0.879 | 0.191 | 1.000 |
|  | 2, O, N | 0.912 | 1.240 | 0.269 | 1.000 |
|  | 3, O, N | 0.670 | 0.911 | 0.198 | 1.000 |
|  | None, M, N | -0.010 | -0.013 | -0.003 | 1.000 |
|  | 1, M, N | 0.884 | 0.907 | 0.261 | 1.000 |
|  | 2, M, N | 0.806 | 0.827 | 0.238 | 1.000 |
|  | 3, M, N | 0.811 | 0.832 | 0.240 | 1.000 |
| 1, O, N | 2, O, N | 0.266 | 0.361 | 0.079 | 1.000 |
|  | 3, O, N | 0.024 | 0.032 | 0.007 | 1.000 |
|  | None, M, N | -0.656 | -0.674 | -0.194 | 1.000 |
|  | 1, M, N | 0.238 | 0.292 | 0.070 | 1.000 |
|  | 2, M, N | 0.160 | 0.164 | 0.047 | 1.000 |
|  | 3, M, N | 0.165 | 0.169 | 0.049 | 1.000 |
| 2, O, N | 3, O, N | -0.242 | -0.329 | -0.072 | 1.000 |
|  | None, M, N | -0.922 | -0.946 | -0.272 | 1.000 |
|  | 1, M, N | -0.028 | -0.029 | -0.008 | 1.000 |
|  | 2, M, N | -0.106 | -0.130 | -0.031 | 1.000 |
|  | 3, M, N | -0.101 | -0.104 | -0.030 | 1.000 |
| 3, O, N | None, M, N | -0.680 | -0.698 | -0.201 | 1.000 |
|  | 1, M, N | 0.214 | 0.220 | 0.063 | 1.000 |
|  | 2, M, N | 0.136 | 0.140 | 0.040 | 1.000 |
|  | 3, M, N | 0.141 | 0.173 | 0.042 | 1.000 |
| None, M, N | 1, M, N | 0.894 | 1.216 | 0.264 | 1.000 |
|  | 2, M, N | 0.816 | 1.110 | 0.241 | 1.000 |
|  | 3, M, N | 0.821 | 1.116 | 0.243 | 1.000 |
| 1, M, N | 2, M, N | -0.078 | -0.106 | -0.023 | 1.000 |
|  | 3, M, N | -0.073 | -0.100 | -0.022 | 1.000 |
| 2, M, N | 3, M, N | 0.005 | 0.007 | 0.001 | 1.000 |

**Table 4. Post hoc comparisons, Experiment 2: learning blocks completed by vocal presence by exposure condition interaction.** Paired-samples t-tests (df = 13) were performed, comparing mean total duration of vocal element perception during the MIDI blocks across levels of the factors of prior learning blocks completed, vocal presence in original excerpts, and exposure condition. *P*-values are Bonferroni corrected for comparing a family of 120. **Legend**: Learning blocks factor: None= no prior learning blocks completed, 1= one learning block completed, 2 = two learning blocks completed, 3 = three learning blocks completed. Exposure condition: O = original excerpt heard during learning block, M = only MIDI-converted version heard during learning block. Vocal presence factor: Y = vocals present in corresponding original excerpt, N = original excerpt contains only instrumental elements.


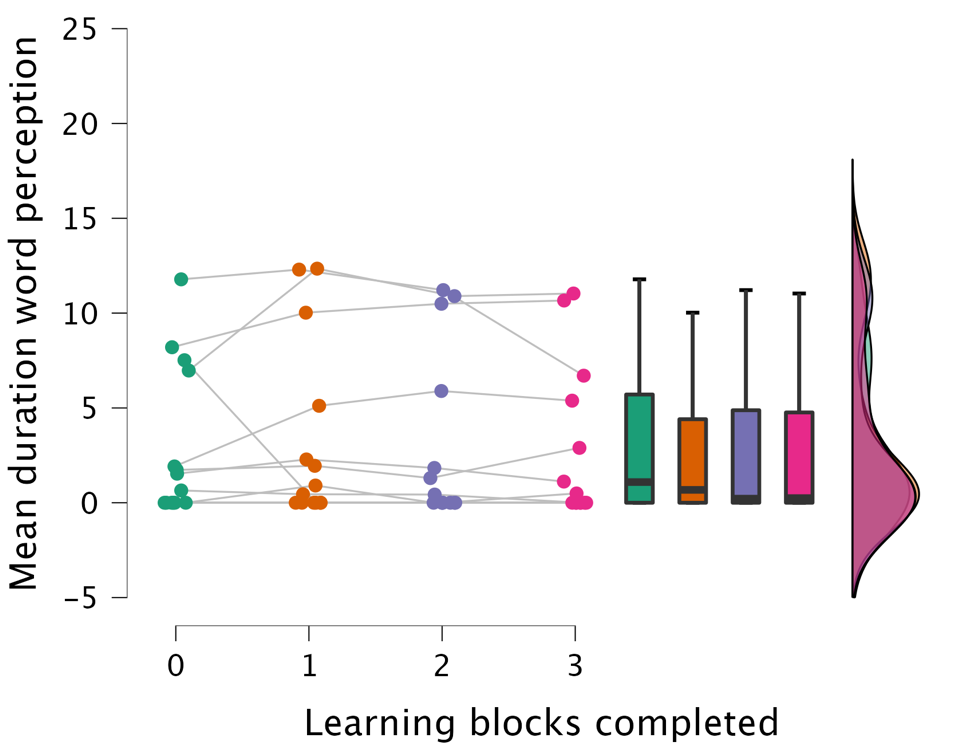


**Figure 1. Mean total duration word perception for unlearned songs, Experiment 2**. For MIDI stimuli containing vocal elements, and corresponding to MIDI-only excerpts heard during learning blocks, mean total duration word perception is shown, with each set of connected dots corresponding to a single participant's data. The x-axis depicts the number of learning blocks completed. Regardless of exposure condition, an increase in the perception of non-specific vocal elements for MIDI stimuli corresponding to original excerpts containing vocal elements was observed. Of the 14 listeners in this experiment, four experienced no perception of words for these stimuli. Box and whisker elements show the mean total duration as a horizontal line, interquartile range is encompassed as the boundaries of the bar, the range as error bars. Distributions associated with each phase are shown on the right. No change in the incidence of word perception for these stimuli was observed across the completion of learning blocks.


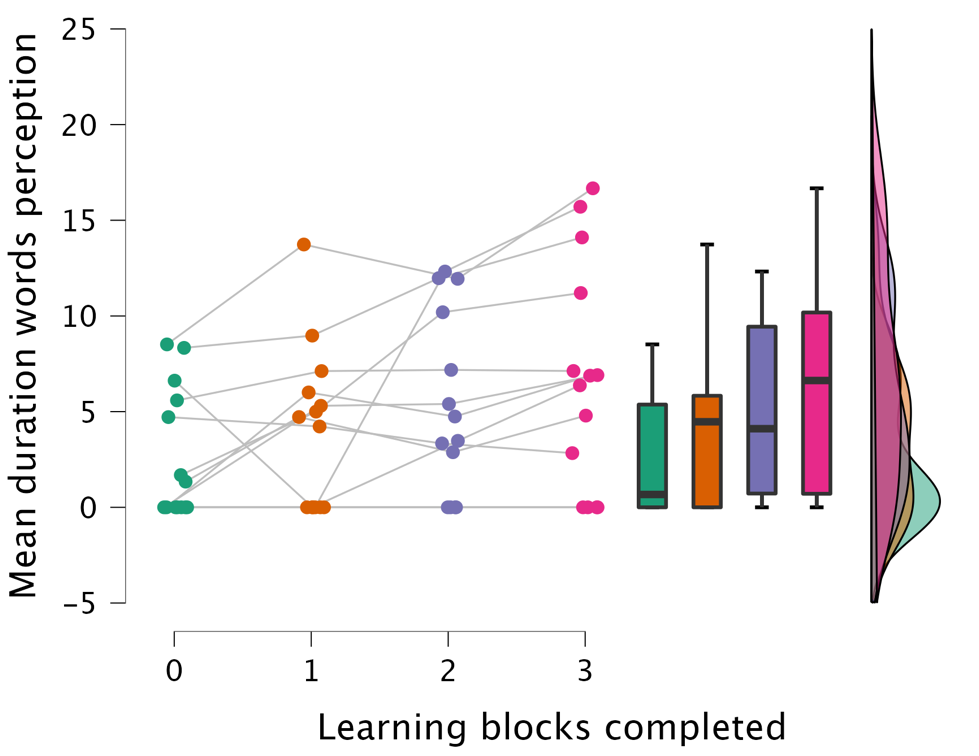


**Figure 2. Mean total duration word perception for learned songs, Experiment 2**. For MIDI stimuli containing vocal elements, and corresponding to original excerpts heard during learning blocks, mean total duration word perception is shown, with each set of connected dots corresponding to a single participant's data. The x-axis depicts the number of learning blocks completed. Regardless of exposure condition, an increase in the perception of non-specific vocal elements for MIDI stimuli corresponding to original excerpts containing vocal elements was observed. Of the 14 listeners in this experiment, three experienced no perception of words at any time for these stimuli. Box and whisker elements show the mean total duration as a horizontal line, interquartile range is encompassed as the boundaries of the bar, the range as error bars. Distributions associated with each phase are shown on the right. Over the course of the experiment, mean duration of perceived words increased for MIDI files containing vocal elements and corresponding to original excerpts heard during learning blocks.


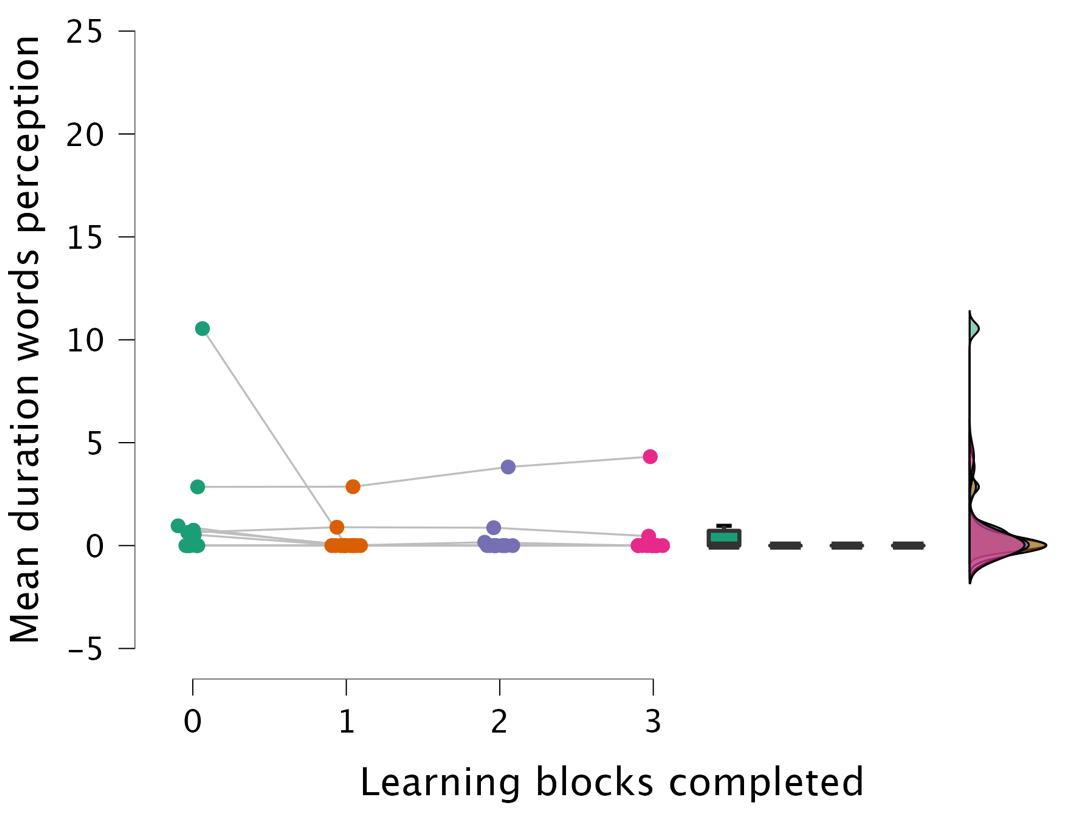


**Figure 3. Mean total duration word perception, Experiment 2**. For MIDI stimuli containing only instrumental elements and presented during the MIDI-only blocks, and corresponding to MIDI-only excerpts heard during learning blocks, mean total duration vocal perception is shown, with each set of connected dots corresponding to a single participant's data. The x-axis depicts the number of learning blocks completed. Of the 14 listeners in this experiment, seven perceived non-specific vocal elements within instrumental-only excerpts at some point during the MIDI-only blocks. Following the completion of three learning blocks, only two participants reported any perception of words during the MIDI-only block. No significant changes in the incidence of the perception of vocal elements for MIDI-converted stimuli containing only instrumental elements was observed over the course of the experiment. Box and whisker elements show the mean total duration as a horizontal line, interquartile range is encompassed by the boundaries of the bar, and error bars depict the range. Distributions associated with each phase are shown on the right.


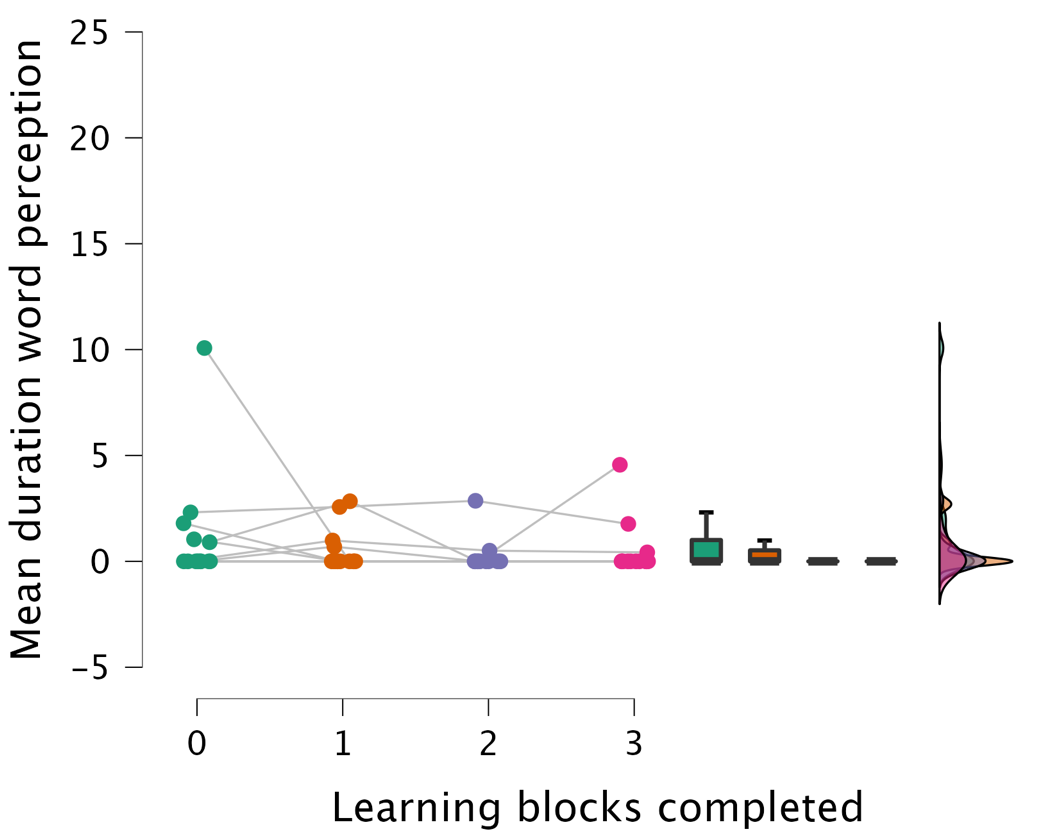


**Figure 4. Mean total duration word perception, Experiment 2**. For MIDI stimuli containing only instrumental elements, and corresponding to original excerpts heard during learning blocks, mean total duration vocal perception is shown, with each set of connected dots corresponding to a single participant's data. The x-axis depicts the number of learning blocks completed. Of the 14 listeners in this experiment, seven perceived non-specific vocal elements within instrumental-only excerpts at some point during the MIDI-only blocks. Following the completion of three learning blocks, only three participants reported any perception of words during the MIDI-only block. No significant changes in the incidence of the perception of vocal elements for MIDI-converted stimuli containing only instrumental elements was observed over the course of the experiment. Box and whisker elements show the mean total duration as a horizontal line, interquartile range is encompassed by the boundaries of the bar, and error bars depict the range. Distributions associated with each phase are shown on the right.
